# Realities of replication: implementation of evidence-based interventions for HIV prevention in real-world settings

**DOI:** 10.1186/1748-5908-9-5

**Published:** 2014-01-06

**Authors:** Shayna D Cunningham, Josefina J Card

**Affiliations:** 1 Yale University, 135 College Street, Room 226, New Haven, CT, 06510, England; 2 Sociometrics Corporation, 201 Main Street, Suite 100, Los Altos, CA 94022, USA

**Keywords:** HIV/AIDS, Evidence-based intervention, Behavioral intervention, Dissemination, Implementation, Adaptation

## Abstract

**Background:**

To have public health impact, evidence-based interventions (EBIs) must be implemented appropriately at meaningful scale. The Center for Disease Control and Prevention’s Replicating Effective Programs and Diffusion of Effective Behavioral Interventions programs disseminate select EBIs by providing program materials and training health providers on their appropriate use and implementation. Sociometrics’ HIV/AIDS Prevention Program Archive (HAPPA) and Program Archive for Sexuality, Health, and Adolescents (PASHA) are likewise the largest EBI collections targeting sexual risk behaviors in the private sector. This study examined the extent to which organizations that obtain EBIs from HAPPA and PASHA implement, adapt and evaluate them and factors associated with program implementation.

**Methods:**

Survey data were collected from 123 organizations that acquired, and had been in possession for a minimum of six months, at least one EBI from HAPPA or PASHA between January 2009 and June 2011. Data regarding program characteristics and date of acquisition were obtained from Sociometrics’ sales and marketing databases. Logistic regression was used to assess barriers to program implementation.

**Results:**

Among organizations that obtained an EBI from Sociometrics intending to implement it, 53% had implemented the program at least once or were in the process of implementing the program for the first time; another 22% were preparing for implementation. Over the three-year time period assessed, over 11,381 individuals participated in these interventions. Almost two-thirds (65%) of implementers made changes to the original program. Common adaptations included: editing content to be more current and of local relevance (81%); adding, deleting or modifying incentives for participation (50%); changing the location in which the program takes place (44%); and/or changing the number, length and/or frequency of program sessions (42%). In total, 80% of implementers monitored program delivery. Participant outcomes were tracked by 78%; 28% of which used evaluation designs that included a control or comparison group. Lack of adequate resources was significantly associated with decreased likelihood of program implementation (odds ratio = 0.180, p <0.05).

**Conclusions:**

Findings provide greater understanding of implementation processes, barriers and facilitators that may be used to develop strategies to increase the appropriate use of EBIs.

## Background

Since the beginning of the epidemic, considerable resources have been spent developing and rigorously evaluating HIV prevention programs. A wide variety of evidence-based interventions (EBIs) have been shown to reduce HIV-related sex and drug risk behaviors among a range of high risk populations. Consequently, over the past several years, government and other funding agencies have expressed a preference for making use of existing, well-tested HIV prevention interventions and approaches ‘that work,’ rather than spending resources on innovation [1-3]. The Centers for Disease Control and Prevention’s Division of HIV/AIDS Prevention (DHAP) includes the implementation of EBIs as part of its strategic plan [4]. EBIs originally developed for HIV prevention are also included on the approved program lists for other health initiatives such as the Office of Adolescent Health (OAH)-administered Teenage Pregnancy Prevention Program, which, in 2010, awarded $75 million in competitive grants to a broad range of organizations and agencies to implement evidence-based programs to prevent teen pregnancy [5]. By replicating effective programs, practitioners operating on limited budgets have a promising blueprint to work with, thereby improving their chances of success.

Several strategies have been developed to promote the translation of efficacious HIV prevention programs into real-world settings. For example, the CDC’s Replicating Effective Programs (REP) and Diffusion of Effective Behavioral Interventions (DEBI) projects provide materials, training, and technical assistance to support implementation of EBIs [6,7]. Although this effort has been proven effective at encouraging organizations to adopt evidence-based programming, the trainings and technical assistance provided are extremely resource-intensive. Sociometrics Corporation has likewise developed the HIV/AIDS Prevention Program Archive (HAPPA) and Program Archive for Sexuality, Health, and Adolescents (PASHA), collections of HIV prevention program packages shown to be efficacious in preventing HIV infection or its risk-related behavioral antecedents among adults and adolescents, respectively [8-10]. Each program package contains everything that a new site would need to implement an EBI, such as a user guide that gives an overview of the program and the evidence of its effectiveness; a facilitator’s manual that gives step-by-step implementation protocols for each session; and session implementation materials referenced in the facilitator’s manual, such as slides, video clips, participant handouts, activity masters, checklists, and homework assignments for the next session. The program packages also contain evaluation materials such as surveys and questionnaires that were used in the original demonstration of effectiveness and that may be used to reevaluate the program as implemented in a new setting. Programs are selected by an independent Scientific Expert Panel based on information available in peer-reviewed journals regarding quality of program implementation; scientific rigor of evaluation; and positive effects on HIV risk behavior, antiretroviral therapy adherence, or biological markers (STI/HIV rates or viral load). Training is available, but not required, to implement the programs. Free technical assistance is provided to all users upon request as needed. Over the past 17 years, Sociometrics has disseminated thousands of HAPPA and PASHA products, generating more than $1.1 million in sales revenues. ETR Associates and other program developers also independently publish and disseminate EBIs that they created.

The availability of replication kits for effective HIV prevention programs facilitates, but does not guarantee, their appropriate use by practitioners [11]. Harshbarger, Simmons, Coelho, Sloop and Collins, for example, found that of the 162 agencies that had obtained and even sent a staff member to be trained to implement a brief, 45-minute, single-session, HIV prevention intervention, only 38% implemented the program within the expected time frame [12]. Moreover, despite recommendations against making substantial changes to pre-packaged EBIs, organizations frequently intentionally modify the content, scope, focus and/or delivery method [13,14]. Such changes may be necessary to improve the fit between the intervention and new target population or context and promote maintenance and sustainability of the program over time, but may also compromise effectiveness.

A growing body of literature has identified a wide variety of reasons why organizations may have difficulty implementing EBIs [15-19]. Among the most prominent of these are factors associated with the organizational contexts into which interventions are introduced, such as organizational capacity, congruence with other agency programs or goals, availability of technical and resource assistance, and ‘buy-in’ among staff and other local stakeholders. Despite their perceived importance, however, few empirical studies have examined how such factors affect implementation and maintenance over time [2].

The overall objective of this study was to examine how EBIs for HIV prevention disseminated via Sociometrics’ HAPPA and PASHA are used in real world settings. Specifically, we assessed: the extent to which these EBIs are implemented; the extent to which the EBIs that are implemented are modified, evaluated and/or sustained over time; and organizational characteristics associated with implementation. This is the first implementation study of EBIs for HIV prevention to assess a private-sector alternative to the CDC’s REP and DEBI dissemination programs.

## Methods

### Participants

We employed a universal sampling scheme to reach all organizations that had obtained at least one EBI via HAPPA or PASHA in the recent past (between January 2009 and June 2011). During this time, a total of 187 organizations obtained 381 program packages for 50 unique programs. Reasons an agency may order multiple program packages include, but are not limited to, that they plan to implement a particular program in multiple sites or that they would like to closely examine materials for a variety of EBIs before selecting which, if any, to implement. A total of 16 (9%) organizations were unable to be contacted, 48 (26%) were successfully contacted but did not participate in the survey because an individual responsible for the program(s) obtained could not be identified or declined to participate, and 123 (66%) completed the survey. Individuals who completed the survey had worked an average of 9.8 years (standard deviation [SD] = 7.5 years) in HIV prevention and included executive directors, program directors, managers, coordinators, and facilitators, health educators, and other prevention specialists. In total, 107 (87%) were female. A total of 86 (70%) self-identified as being White, 25 (20%) as Black, 10 (8%) as Hispanic, 1 (1%) as Asian, and 1 (1%) as mixed race/ethnicity.

### Procedures

A minimum of five phone call attempts were made and two emails sent to reach an eligible representative from each organization. Eligibility requirements included being 18 years of age or older and responsibility for the selection and/or implementation the HIV prevention program package(s) obtained from Sociometrics. All organizations had been in possession of their selected EBI(s) for six months to almost three years prior to being contacted.

Survey data were collected from September to December 2011 via SurveyMonkey. Items assessed included: organizational characteristics such as type and main activities of the organization, type of area served, extent to which HIV prevention is a priority, number of paid full-time equivalent staff, number of paid full-time equivalent staff who work specifically in HIV prevention, and implementation of other EBIs; reasons for EBI acquisition; extent to which program implementation was achieved; and level of agreement (1 = strongly disagree to 5 = strongly agree) regarding six factors posited to influence program implementation, including lack of program alignment with the needs of local population and/or setting, poor program fit with other agency programs or goals, lack of availability of funding and other resources, absence of a ‘champion’ committed to program implementation, lack of organizational support for the program, and lack of support from local stakeholders. For each factor, responses were dichotomized as ‘yes’ or ‘no’ that it was a barrier to program implementation. Participants representing organizations that had implemented or were currently implementing the program were additionally asked a series of yes or no questions regarding: characteristics of the target population reached (*i.e*., number, age, sex, race/ethnicity, sexual orientation, HIV status, and substance use behaviors of persons enrolled in the program); type of adaptations made (*e.g*., translated materials into another language, edited content to be more current and of local relevance, added activities, removed activities); reasons for program adaptations made (*e.g*., to make the program more acceptable to the target population, to increase the relevance and compatibility of the program with the implementing agency’s mission, to increase ‘buy-in’ for the program among other local stakeholders, to make it more feasible to implement the program given resource constraints); process evaluation activities conducted (*e.g.*, tracked program participation, assessed participant satisfaction with the program, assessed staff attitudes toward program, assessed fidelity of program implementation); outcome evaluation activities conducted (*e.g*., types of surveys conducted, whether comparison or control groups were included); and training and technical assistance received (*e.g*., general training on program implementation, training on how to implement or help implementing a specific program, general training on how to adapt evidence-based programs, training on how to adapt or help adapting a specific program, general training on program evaluation, training on how to evaluate or help evaluate a specific program). Data regarding characteristics of the EBIs such as type (*e.g*., one-on-one, small group, or community-wide), duration (*e.g*., single versus multi-session), original implementation setting (*e.g*., clinic-, community-, or school-based), program package format (*e.g*., download versus hard-copy), and number of months organizations were in possession of the program prior to completing the survey, were obtained from Sociometrics’ sales and marketing databases.

Respondents completed the survey for only one program obtained per organization. For organizations that had obtained multiple programs (as opposed to multiple copies of the same program), study staff randomly selected the program about which the participant completed the survey. The surveys lasted approximately 10 minutes to 30 minutes, depending on whether the organization had ever implemented the program obtained. Survey participants were compensated $25 for their time, either as a personal check or donation to their organization. All of the procedures were approved by Sociometrics’ Institutional Review Board.

### Data analyses

All analyses were conducted using SPSS 17.0 (SPSS Inc, Chicago, IL). Initial descriptive analyses were performed to examine the extent to which program implementation was achieved as well as reasons for not implementing the EBI obtained. Statistical analyses were performed in a step-wise fashion using logistic regression with 95% confidence intervals for each odds ratio. First, we tested whether various organizational and program characteristics were associated with whether program implementation had occurred. All of the variables that were significantly associated with program implementation were then entered in a single block. All analyses controlled for length of time an organization had been in possession of the program prior to completing the survey. Statistical significance was defined as p <0.05.

## Results

### Organizational and intervention characteristics

Table 1 summarizes the characteristics of the 123 organizations studied in this research and the EBIs they obtained. Nearly half (46%) were community-based organizations, and more than three-fourths (78%) included direct service delivery as one of their main activities. Most served urban areas (90%), although a large proportion exclusively targeted or included rural settings as part of their target populations (11% and 57% respectively). A broad range of small and large organizations were represented, 58% of which considered HIV prevention a high priority, and 54% of which were implementing other EBIs for HIV prevention.

**Table 1:** Organizational and program characteristics (N = 123)

**Characteristic**	**N (%)**
**Organizational**	
Type of organization^a^	
Local/national NGO	20 (16)
International NGO	1 (1)
Faith-based organization	3 (2)
Community-based organization	57 (46)
Network alliance/umbrella organization	4 (3)
Research institute/think-tank	7 (6)
Academic institution	20 (16)
Other (*e.g*., health department, detention center, school health program )	28 (23)
Main activities^a^	
Service delivery	94 (76)
Advocacy/networking	57 (46)
Research	19 (15)
Other	41 (33)
Area served	
Rural	13 (11)
Urban	40 (33)
Both rural and urban	70 (57)
Priority of HIV prevention	
Not a priority	3 (2)
A low priority	13 (11)
A medium priority	34 (28)
A high priority	71 (58)
Don’t know/No response	2 (2)
Number of paid full-time staff	
0	3 (3)
1-5	21 (17)
6-10	15 (12)
11-15	8 (7)
16-20	9 (7)
21-25	6 (5)
25+	60 (49)
Don’t know/No response	1 (1)
Number of full-time staff devoted to HIV prevention	
0	22 (18)
1-5	52 (42)
6-10	14 (11)
11-15	10 (8)
16-20	5 (4)
21-25	2 (2)
25+	17 (14)
Don’t know/No response	1 (1)
Implementing other EBI for HIV prevention	
Yes	64 (54)
No	53 (43)
Don’t know/No response	6 (5)
**Program**	
Type	
One-on-one	27 (22)
Small group	94 (76)
Community-wide	2 (2)
Duration	
Single session	9 (7)
Multi-session	114 (93)
Original implementation setting	
Clinic-based	48 (39)
Community-based	49 (40)
School-based	26 (21)
Program package format	
Download	70 (57)
Hard-copy	53 (43)

^a^Categories are not mutually exclusive.

The majority of EBIs obtained by organizations included in the study were small-group (76%), multi-session (93%) programs, originally implemented in community-based (40%) or clinic-based (39%) settings. A total of 57% of the program packages for these EBIs were obtained from Sociometrics in a downloadable, versus hard-copy, format. On average, organizations had been in possession of their EBIs for 16.4 (SD = 8.1) months.

### Program implementation

A total of 45 (37%) organizations had neither implemented their selected EBI nor were planning to do so. Of these, 19 (42%) had not ever intended to implement the program. Rather, commonly cited reasons for EBI acquisition included: to obtain information about the program for a grant application or research proposal (11%); to use activities from the intervention as part of another program offered (5%); to help inform the development of a new program (11%); and/or to provide training or technical assistance to others implementing the program (42%).

Among organizations that obtained their selected EBI with the intent to implement it (N = 104), 55 (53%) had implemented the program at least once or were implementing it for the first time (30% and 23%, respectively), and another 23 (22%) were in the process of preparing for implementation. Among those that had implemented their selected EBI at least once (N = 31), 68% had done so more than once, and 48% were still offering the program.

### Program reach

EBIs obtained by organizations surveyed for this study had been implemented with more than 11,381 individuals. Table 2 lists the settings in which program implementation occurred and describes EBI participant characteristics. A total of 58% of organizations that implemented the EBI they obtained (N = 55) did so at community-based organizations. Other settings in which program implementation occurred included: schools (25%); clinics, hospitals, and/or treatment facilities (24%); community-wide (22%); correctional facilities (11%); and/or other locations (9%) such as faith-based organizations and apartment complexes. Programs were most commonly implemented with adolescent females (78%) followed by adolescent males (45%), adult females (22%), and adult males (15%). EBI participants at 87% and 67% of organizations included persons who were Black or African American and Hispanic, respectively. Nearly all (93%) organizations implemented their respective programs with persons who were heterosexual, while 58%, 44%, and 27% of organizations’ EBI participants also or alternatively included persons who were bisexual, lesbian, and/or gay/men who have sex with men (MSM), respectively. Approximately half of the implementing organizations (60%) did not know the HIV status of their EBI participants; 24% reported that their programming efforts included individuals who were HIV positive. Few (2%) specifically targeted persons living with HIV (PLH).

**Table 2:** EBI implementation setting and participant characteristics (N = 55)

	**N (%)**
**Implementation Setting**	
Locale^a^	
Clinic/hospital/treatment facility	13 (24)
Community-based organization	32 (58)
Community-wide (neighborhood/city/county/etc.)	12 (22)
Correctional facility	6 (11)
School	14 (25)
Other	5 (9)
**Participant Characteristics**	
Age^a^	
Adolescent males	25 (45)
Adolescent females	43 (78)
Adult males	8 (15)
Adult females	12 (22)
Race/Ethnicity^a^	
Black or African American	48 (87)
Hispanic/Latino	37 (67)
White	32 (58)
Asian	16 (29)
Native Hawaiian or other Pacific Islander	7 (13)
American Indian/Alaska Native	12 (22)
Sexual Orientation^a^	
Gay/Men who have sex with men (MSM)	15 (27)
Lesbian	24 (44)
Bisexual	32 (58)
Heterosexual	51 (93)
HIV Status	
Negative	9 (16)
Positive	1 (2)
Both negative and positive	12 (22)
Unknown	33 (60)

^a^Categories are not mutually exclusive.

### Program adaptations

Among organizations that implemented the EBI they obtained (N = 55), 36 (65%) reported having made changes to the original content and design. Table 3 lists the types of and reasons for program changes that were made. The most common adaptations included both minor (*e.g*., updating information presented to be more current and of local relevance; adding, deleting, or modifying incentives for participation; changing the location in which the program takes place) and more substantial (*e.g*., changing the number, length, and/or duration of sessions) changes to the EBIs’ original content and designs. A total of 78% of organizations that made such changes did so to increase the relevance, acceptability, or appeal of the program to the target population (78%), followed by 47%, 22%, and 19% that did so to increase the feasibility to implement it given resource constraints, relevance and compatibility of the program with their mission, and/or ‘buy-in’ from other local stakeholders, respectively.

**Table 3:** **Types of and reasons for changes made to EBIs**^**a **^**(N = 36)**

	**N (%)**
**Types of Adaptations**	
Translated program materials into another language	5 (14)
Edited content to be more current and of local relevance	29 (81)
Changing the number, length, and/or frequency of program sessions	15 (42)
Adding activities without removing any of the original ones	9 (25)
Deleting activities without adding any new ones	4 (11)
Substituting activities	10 (28)
Adding, deleting, or modifying incentives for participation	18 (50)
Changing the location in which the program takes place (*e.g*., from a clinic to a community-based organization)	16 (44)
**Reasons for adaptations**	
To make the program more relevant, acceptable, or appealing to the target population	28 (78)
To increase the relevance and compatibility of the program with the implementing agency’s mission	8 (22)
To increase ‘buy-in’ for the program among other local stakeholders	7 (19)
To make it more feasible to implement the program given resource constraints	17 (47)

^a^Categories are not mutually exclusive.

### Evaluation activities

Among organizations that implemented the EBI they obtained (N = 55), 44 (80%) had conducted or were conducting one or more of the following process evaluation activities: tracking program participation (84%); assessing participant satisfaction with the program (86%); assessing staff attitudes toward the program (66%); and assessing fidelity of program implementation (64%). In total, 43 (78%) had assessed and/or were tracking participant outcomes such as changes in participant knowledge, attitudes or behaviors. Study designs for these assessments included: post-test survey only (5%); pre-test and post-test surveys (53%); and pre-test, post-test, and follow-up surveys (42%). A total of 28% included a control or comparison group. Among organizations that reported having made adaptations to the EBI they implemented or were currently implementing (N = 36), 89% and 75% assessed or were monitoring program delivery and/or assessing participant outcomes, respectively.

### Factors associated with program implementation

None of the organizational or program characteristics listed in Table 1 were significantly associated with whether program implementation took place. Table 4 shows the relationship between other factors posited to be related to organizational context and whether program implementation occurred, among organizations that obtained their selected EBI with the intent to implement it (N = 104).

**Table 4:** Odds ratios for potential factors associated with program implementation

**Barrier**	**Organization implemented or is currently implementing program, N (%)**		**Odds ratio**^**b**^	
	**Yes (N = 55)**	**No**^**a **^**(N = 49)**	**Unadjusted**	**Adjusted**
Program did not meet (and could not be easily adapted to meet) the needs of the local population and/or setting.	3 (5)	10 (20)	0.213*	0.258
Program is not a good fit (and cannot be easily adapted to be a good fit) with other agency programs or goals.	6 (11)	10 (20)	0.460	--
Lack of adequate funding, staff, and other resources are available to implement the program.	9 (16)	26 (53)	0.171*	0.180*
Lack of a ‘champion’ committed to implementing the program.	5 (9)	12 (24)	0.307*	1.1
Lack of organizational support for the program.	3 (5)	8 (16)	0.296	--
Lack of support from local stakeholders.	4 (7)	10 (20)	0.303	--

^a^Sample includes only organizations that obtained an EBI with the intent to implement it.

^b^Controlling for number of months organizations were in possession of their respective EBIs.

*p <0.05

In bivariate analysis, program implementation having not occurred was associated with a lack program alignment with the needs of local population and/or setting; lack of adequate funding, staff, and other resources; and lack of the presence of a ‘champion’ committed to program implementation. In multivariable analysis, only lack of adequate funding, staff, and other resources remained independently associated with decreased likelihood of organizations having implemented the EBI they obtained (odds ratio = 0.180, p <0.05).

Among program implementers (N = 55), 85% reported having received a grant or donation to fund the program. Only 5% reported that no additional funds were needed for program implementation; rather, the EBI had been integrated into other ongoing initiatives, programs or activities. The remainder did not specify how program implementation had been or was being supported.

### Training and technical assistance received

Among organizations that implemented the EBI they obtained (N = 55), 76% reported having received training or technical assistance with program implementation and/or evaluation activities including: training on program implementation in general (45%); training on how to implement or help implement a specific program (47%); training on how to adapt EBIs in general (47%); training on how to adapt or help adapt a specific program (33%); training on program evaluation in general (45%); and/or training on how to evaluate or help evaluate a specific program (31%). Likewise, among organizations that were planning for but had not yet begun program implementation (N = 23), 65% reported that staff had received or would receive training and/or technical assistance in one or more of the areas described above.

## Discussion

In order to maximize their public health impact and successfully change behavior among diverse populations, EBIs must be widely disseminated, appropriately implemented, and sustained over time. Our findings indicate that organizations may obtain materials for evidence-based HIV prevention programs for a variety of reasons, including: to implement the program, to obtain information for a grant application or research proposal, and/or to provide training or technical assistance to others implementing the program. Among those who obtained EBIs with the intent to implement them, a majority (53%) had implemented the program at least once or were in the middle of implementing it for the first time. Almost another quarter (22%) were in the preimplementation phase (*i.e*., preparing for implementation). Thus, a total of 75% of organizations were actively working with their respective programs within a minimum of six months of having obtained them. Moreover, many organizations that obtained EBIs via HAPPA and PASHA have successfully been able to sustain program implementation over time.

These rates of adoption are consistent with, and in some cases higher than, those reported for DEBI programs [12,20], despite attendance at program-specific training not being a requirement to obtain intervention materials from Sociometrics. Having received training is associated with an increased likelihood of AIDS services organizations offering EBIs to their clients [18]. Nearly half of organizations that had implemented or were currently implementing the EBI they obtained via HAPPA or PASHA reported that they had received training on or technical assistance with program implementation in general (45%) and/or for their specific EBI (47%). Training is not mandated to acquire programs from these collections; thus, this finding indicates that many sites will take the initiative to seek such assistance on their own.

Within the three-year study time frame, EBIs obtained from Sociometrics were implemented with a diverse pool of over 11,300 individuals. The target populations for the majority of these programs included blacks and/or Hispanics, groups disproportionately affected by HIV and who have been a priority for new EBI development [21]. Adolescents were the most common age group targeted, particularly adolescent females, likely due to several current funding streams focused on teen pregnancy prevention that include EBIs disseminated via PASHA on their lists of approved programs such as the OAH-sponsored Teen Pregnancy Prevention Program [5]. Another high priority population exposed to over a quarter of the EBIs implemented by organizations assessed in this study was gay men, including men who have sex with men (MSM) who may or may not self-identify as gay. Few programs that were implemented or being implemented specifically targeted PLH. An increased number of evidence-based secondary HIV prevention programs have become available in recent years. Coupled with increasing efforts to include behavioral interventions for PLH as part of more comprehensive approaches to HIV prevention [4,21], the proportion of organizations implementing such programs is expected to grow.

Our finding that almost two-thirds (65%) of program implementers had made changes to the original content and/or design is consistent with previous implementation studies of DEBI programs, where adaptations were found to be commonplace [12,20,22,23]. The rationale for these changes was likewise similar, with the most commonly cited reasons being to make the program more relevant, acceptable or appealing to the target population and/or more feasible to implement given resources constraints. The opportunity to make adaptations may also enable agency staff to develop a sense of ownership of a program [13].

The most common type of changes reported by study participants were editing content to be more current and of local relevance, and adding, deleting or modifying incentives for participation. These are relatively minor, and generally considered allowable, adaptations. Conversely, changing the number, length and/or frequency of program sessions, substituting activities, and/or deleting activities (adaptations reported by 42%, 28%, and 11% of participating organizations that had implemented or were implementing the EBIs they obtained, respectively) are much more likely to threaten program fidelity and, in turn, potentially negatively affect desired outcomes. The ADAPT and ADAPT-ITT frameworks are logic models that detail the steps involved in organizations’ adoption, adaptation and implementation of EBIs [13,24]. There is a recognized need, however, for more explicit guidance regarding how specific programs can and cannot be adapted during translation [13].

In recent decades, the nonprofit sector has faced increasing pressures of accountability for program success, with an emphasis on measuring outcomes and impacts rather than just activities and inputs [25]. Many funders, for example, now require detailed evaluation plans as part of proposal applications. Most organizations (80%) that implemented the EBI they obtained actively monitored program delivery, including tracking program participation, assessing participant satisfaction with and staff attitudes toward the program, and/or, to a slightly lesser extent, assessing fidelity of program implementation. A similarly large proportion (78%) had assessed and/or was tracking participant outcomes such as changes in participant knowledge, attitudes and behaviors. Only a small minority of these (28%) included a control or comparison group. HAPPA and PASHA are marketed as evidence-based products for service providers. Service providers who implement EBIs should conduct outcome monitoring to determine whether a reduction in risk behaviors occurs among their clients that participate. This is especially true if substantial changes were made to the program. Determining whether risk behavior change is fully attributable to the adapted intervention would require a more complex evaluation design using some comparison condition, but this is often beyond the capacity of many direct HIV prevention service providers. Other research has also shown that agencies seldom pre-test or pilot EBIs to which they have made changes prior to implementation [13].

Although use of EBIs is considered to be a more cost-effective approach to HIV prevention than developing new programs, of the organizational characteristics assessed in this study, availability of funding, staff, and other resources was nonetheless the only factor found to be independently significantly associated with whether program implementation took place. For most (85%) of the organizations that had achieved program implementation, this work was supported by an outside grant or donation. Given funding to implement an EBI, two other important factors associated with program implementation are the goodness of fit between the intervention and needs of the local context, and the presence of a program champion. The relationship between other factors associated with organizational context not assessed in this study (*e.g*., culture, climate, work attitudes) and program implementation should be the focus of future research [2]. In particular, it would be beneficial for future work to investigate how these organizational contextual factors might facilitate successful implementation and maintenance of EBIs as part of their services, absent additional funding.

There were several limitations to this study. Participants represented only 66% of eligible organizations; thus, caution should be exercised when generalizing the findings. Selection bias among the respondents may have led to a skewed picture of EBI implementation. This study also relied on self-reported, rather than objective, measures of program implementation, adaptation, and evaluation activities, thus may be subject to recall and/or social desirability biases. We attempted to mitigate the former by limiting our sample to include only organizations that had obtained their respective EBIs in the past three years. Likewise, the selection criteria for respondents included that they have been involved in the review, selection, implementation, and evaluation of evidence-based programs at their respective organizations and were familiar with the program package that was obtained from Sociometrics. It is possible, however, that staff turnover may have affected the reliability of some respondents’ answers (*e.g*., their knowledge of reasons for EBI acquisition if they were not on staff at that time). Although the survey asked about attempts at evaluation, we do not know what measures were assessed or how many of these efforts were able to document significant changes. Owing to the cross-sectional nature of this research, we also cannot make causal interpretations regarding factors that influence program implementation. The study procedures controlled for the influence of agencies that purchased more than one type of program package. It is a good strategy for organizations to consider multiple options and choose that which is the best fit for their contexts. By randomly selecting the program about which representative from these agencies completed the survey, in some cases, it might appear that an agency had failed to implement an EBI when in fact stakeholders may have made a thoughtful determination to implement an alternative one.

## Conclusions

The decision to acquire a program is distinct from the decision to implement it and how it gets implemented. When translating EBIs to real world settings, challenges are to be expected. Despite its limitations, this study demonstrates the success of Sociometrics’ program archives to promote the adoption, implementation and evaluation of a wide variety of evidence-based HIV prevention programs among a diverse pool of practitioners nationwide. Moreover, its findings provide greater understanding of implementation processes, barriers and facilitators that may be used to develop strategies to increase the use of EBIs.

## Competing interests

Both authors were employed by Sociometrics Corporation at the time the research was conducted.

## Authors’ contributions

SDC conceptualized the research design, conducted the interviews and analyses, and served as the lead writer. JJC supervised all aspects of the study and assisted with writing. Both authors have read and approved the final manuscript.
